# SPatiotemporal-ENcoded acoustic radiation force imaging of focused ultrasound

**DOI:** 10.3389/fnhum.2023.1184629

**Published:** 2023-04-27

**Authors:** Xu Qi, Jiayu Sun, Jiayu Zhu, Dechen Kong, Neil Roberts, Yijing Dong, Xiaoqi Huang, Qiang He, Haoyang Xing, Qiyong Gong

**Affiliations:** ^1^College of Physics, Sichuan University, Chengdu, China; ^2^Huaxi MR Research Center (HMRRC), Department of Radiology, West China Hospital of Sichuan University, Chengdu, China; ^3^Department of Radiology, West China Hospital of Sichuan University, Chengdu, China; ^4^Central Research Institute, United Imaging Healthcare Group, Shanghai, China; ^5^Edinburgh Imaging and Centre for Reproductive Health (CFRH), Queen's Medical Research Institute, University of Edinburgh, Edinburgh, United Kingdom; ^6^Research Unit of Psychoradiology, Chinese Academy of Medical Sciences, Chengdu, China; ^7^Brain Laboratory, United Imaging Research Institute of Innovative Medical Equipment, Shenzhen, China

**Keywords:** acoustic radiation force imaging (ARFI), SPatiotemporal-ENcoded (SPEN), frequency-swept (chirp) pulse, magnetic resonance, focused ultrasound (FU) neuromodulation

## Abstract

Neuromodulation technology has provided novel therapeutic approaches for diseases caused by neural circuit dysfunction. Transcranial focused ultrasound (FU) is an emerging neuromodulation approach that combines noninvasiveness with relatively sharp focus, even in deep brain regions. It has numerous advantages such as high precision and good safety in neuromodulation, allowing for modulation of both peripheral and central nervous systems. To ensure accurate treatment targeting in FU neuromodulation, a magnetic resonance acoustic radiation force imaging (MR-ARFI) sequence is crucial for the visualization of the focal point. Currently, the commonly used 2D Spin Echo ARFI (2D SE-ARFI) sequence suffers from the long acquisition time, while the echo planar imaging ARFI (EPI-ARFI) sequence with a shorter acquisition time is vulnerable to the magnetic field inhomogeneities. To address these problems, we proposed a spatiotemporal-encoded acoustic radiation force imaging sequence (i.e., SE-SPEN-ARFI, shortened to SPEN-ARFI) in this study. The displacement at the focal spot obtained was highly consistent with that of the SE-ARFI sequence. Our research shows that SPEN-ARFI allows for rapid image acquisition and has less image distortions even under great field inhomogeneities. Therefore, a SPEN-ARFI sequence is a practical alternative for the treatment planning in ultrasound neuromodulation.

## 1. Introduction

Ultrasound neuromodulation has been successfully applied to modulate the neural functions, enabling the study of brain function and treatment of brain disorders noninvasively. Recent studies have reported the successful applications of ultrasound neuromodulation in various brain regions, such as the visual cortex (Lee et al., 2016), somatosensory cortex (Legon et al., 2014), motor cortex (Legon et al., 2018), and thalamus, without adverse side effects (Ai et al., 2018). The emerging technique has been used to treat Parkinson's disease (Magara et al., 2014), epilepsy (Lipsman et al., 2013), depression (Nuttin et al., 2014), and other diseases (Leinenga et al., 2016). To ensure safe and accurate application of FU therapy, it is crucial to closely monitor the treatment targets. Magnetic resonance imaging (MRI) is widely used for this purpose to ensure therapeutic efficacy and safety since 1992 (Cline et al., 1992). During MR-guided focused ultrasound (MRgFU) treatment, MRI can monitor temperature and dynamically locate the focus in a real-time way. In particular, the MR acoustic radiation force imaging (MR-ARFI) can visualize the ultrasound focal point *in vivo* by encoding the micro-scale tissue displacement produced by the acoustic radiation pulses in millisecond duration through phase contrast MRI (Holbrook et al., 2011; Qiao et al., 2021). However, traditional MR-ARFI based on spin-echo sequence (i.e., SE-ARFI) has long acquisition times (Kaye and Chen, 2011), typically taking 5–10 min to obtain high-resolution images. Imaging sequences with a faster readout method such as echo-planar imaging (Kaye and Chen, 2011) or spiral (Ilovitsh et al., 2019) have been proposed to accomplish the acquisition within single or few scans. However, these advanced sequences are vulnerable to field inhomogeneities, which is a common problem in the ultrasound applications where the ultrasound transducer would bring in large susceptibility differences. To address these problems, ARFI based on spatiotemporal encoded (SPEN) imaging was proposed as a method to monitor tissue displacement caused by acoustic radiation force during FU therapy. Our results show that SPEN-ARFI is effective in monitoring the effect of FU on both agar phantoms and *ex vivo* porcine brains.

## 2. Materials and methods

We are accountable for all aspects of the study including full data access, integrity of the data, and the accuracy of the data analysis, in ensuring that questions related to the accuracy or integrity of any part of the study are appropriately investigated and resolved.

### 2.1. SPEN MR imaging

Spatiotemporal-encoded magnetic resonance imaging (SPEN-MRI) is a novel ultrafast single-scan MRI technique that has evolved from EPI in recent years. In SPEN-MRI, the traditional RF pulse used to excite spins is replaced by a linear chirp pulse, and a spatiotemporal encoding gradient is applied in the phase encoding direction. As a result, the phase profile of the spins has a quadratic shape, which increases the bandwidth of acquisition gradient in the phase encoding direction. Only the phase stable point and its adjacent area contribute to the SPEN-MRI signal, which reduces chemical shift artifacts and distortions caused by inhomogeneous magnetic fields, as compared to traditional SE-EPI or GRE-EPI images. Similar to SE-EPI, SPEN-MRI requires continuous application of fast-switching magnetic field gradients, but SPEN-MRI uses a special chirp pulse (Shrot and Frydman, 2005).

### 2.2. Chirp pulse

The linear scanning pulse in SPEN-MRI produces a parabolic phase profile. Unlike RF pulses traditionally used in MRI sequences with a fixed central frequency, the central frequency of the chirp pulse used in SPEN-MRI sequences changes linearly with time, producing quadratic phase modulation (Garwood and DelaBarre, 2001; Schmidt and Frydman, 2014). If the frequency bandwidth of the chirp pulse is ΔO, the duration is Te, the spatiotemporal encoding gradient is G_e_, the initial frequency of the excitation is O_i_, and the excitation frequency at time t is ω_c_(t), then


(1) $$\omega_{c}(t)=O_{i}+Rt$$

where R = ΔO/T represents the rate of change of the frequency of the chirp pulse. The phase that is accumulated during the excitation *Φ*_c_(t) can then be expressed as


(2) $$\phi_{c}(t)=\int_{0}^{t}\omega_{c}(t^{\prime })dt^{\prime }$$

and the magnetic field generated by the chirp pulse can be expressed as


(3) $$B(t)=B_{1}(t)[cos(\phi_{c}(t))\hat{x}+sin(\phi_{c}(t))\hat{y}]$$

the intrinsic spatial resolution of SPEN-MRI △*y* (Ben Eliezer et al., 2010) can be expressed as


(4) $$\text{Δ}y=\sqrt{\frac{L_{y}}{\gamma G_{e}T_{e}}}$$

where L_y_ is the FOV size in y direction.

Typically, the amplitude of the chirp pulse is modulated by a square wave function. However, this has the disadvantage that the frequency spectrum contains substantial oscillations, resulting in uneven excitation and artifacts. This led Kupce and Freeman (1996) to propose modulating the amplitude of the chirp pulse by a so-called WURST function.

### 2.3. Displacement encoding gradient

The incorporation of a Displacement Encoding Gradient (DEG) is crucial for SPEN-ARFI imaging (Souchon et al., 2008), with detection sensitivity being adjustable through the adjustment of DEG amplitude and duration. Since SPEN incorporates a 180°RF pulse (Tal and Frydman, 2010), it is well-suited for the use of a bipolar DEG (Mcdannold and Maier, 2008; Chen et al., 2010), with a repetitive design being employed due to the shortest time of the ultrasound pulse. To account for tissue viscoelasticity, a delay time (t_delay_) between the opening time of FU and the application of DEG was investigated to maximize the displacement encoding. The averaged displacement Δx corresponding to the DEG can be obtained according to the following equation


(5) $$\text{Δ}x=\frac{\text{Δ}\varphi }{2\gamma \int_{0}^{\tau }\vec{DEG}(t^{\prime })dt^{\prime }}$$

where Δx is the measured displacement, Δφ represents the phase difference between two phase images collected before and after application of the acoustic radiation force, and τ refers to DEG duration (Xu et al., 2019; Qingpu et al., 2020).

### 2.4. Experimental setup

All of the experiments were conducted in a whole-body 3.0T MRI system [*uMR*790, United Imaging Healthcare (UIH), Shanghai, China] with a 32-channel RF head coil. The FU transducer used to induce local displacements was an 800 kHz single-element design (Imasonic, Besancon, France) with a focal depth of 5 cm. The instantaneous input electrical power used to generate the ARF was set to 170 Watts. For both agar phantom and *ex vivo* porcine brain, the ultrasound transducer was fixed above the object to be scanned, the duration of the FU ARF pulse was 21.76 ms, and images were acquired with the FU system turned on and off.

First, we aimed to evaluate the imaging quality of the SPEN sequence in comparison to commonly used sequences by scanning the same slice of a water phantom with a FU transducer, the parameters used in this experiment are detailed in Table 1. Second, an agar phantom was utilized to test the optimal delay time for the SPEN-ARFI sequence. By setting different delay times and comparing the resulting phase differences, we were able to determine the optimal delay time for the following experiments. The delay time varied in the range of −6–2 ms in optimal delay time experiment and fixed to −3 ms in other experiments. A multi-slice series of the agar phantom and *ex vivo* porcine brain images was acquired in the coronal orientation to determine the location of the target, followed by acquiring images through the target in the axial orientation. Since imaging times were short (125 ms per slice), phase changes that may be potentially produced by temperature changes were insignificant. The resistance of SPEN-ARFI and EPI-ARFI sequences to inhomogeneous magnetic fields was then assessed using the same parameters, and the image distortions were visually evaluated. Finally, the quantitative comparison experiment of SPEN-ARFI and SE-ARFI sequence was conducted using the *ex vivo* porcine brain, the same slice was scanned, and the FU parameters were kept consistent throughout the experiment. The maximum displacement was determined by calculating the average phase difference of nine pixels at the focal point, and the resulting average values obtained by the SE-ARFI sequence and SPEN-ARFI sequence were compared.

**Table 1:** Parameters of the agar phantom comparison experiment.

**Sequence**	**TR**	**TE**	**FOV**	**Acquisition matrix**	**Flip angle**
FSE	5,400 ms	102.9 ms	216 × 216 mm	144 × 129	90°
GRE-EPI	1,000 ms	100 ms	200 × 200 mm	108 × 98	12°
SE-EPI	1,000 ms	100 ms	200 × 200 mm	64 × 64	90°
SPEN	1,000 ms	55–125 ms	200 × 200 mm	64 × 64	90°

The SPEN-ARFI sequence parameters used in agar phantom and *ex vivo* porcine brain imaging were kept consistent, TE1 55 ms, TE2 125 ms, TE ranging from 55 to 125 ms, chirp pulse duration T_e_ 20 ms and bandwidth 30.7 kHz, G_e_ 3 mT/m, DEG 50 mT/m, DEG duration 6 ms, slice number 8, and spacing between slices 7.5 mm. The SPEN-ARFI, EPI-ARFI, and SE-ARFI in this study used these common parameters: field of view (FOV) 200 × 200 mm, flip angle 90°, slice thickness 5 mm, TR 1,000 ms, and acquisition matrix 64 × 64. The TE of SE-ARFI was 50 ms, and the TE of EPI-ARFI was 100 ms in the comparison experiments.

### 2.5. Image reconstruction

Image analysis was performed by using MATLAB 2018b software running on a PC computer equipped with an Intel(R) Core (TM) i7-10875H CPU @ 2.30 GHz and a Nvidia GeForce RTX 2060 4GB. Images were reconstructed offline from the K-space data sampled in Cartesian coordinates by using programs coded in MATLAB software. To obtain the baseline phase information, we calculated and compensated for the quadratic phase according to the corresponding chirp pulse parameters during image reconstruction. The signal S(t_a_) in the y direction acquired at time t_a_ (Zhang et al., 2016) is


(6) $$S(t_{a})=\int_{\frac{-L_{y}}{2}}^{\frac{L_{y}}{2}}\rho_{0}(y)\exp\left[i(-\frac{BW}{2L_{y}}y^{2}+\frac{BW}{2}y+\gamma G_{a}yt_{a})\right]dy$$

The position-related quadratic term can be removed by the following equation


(7) $$\begin{array}{l} S^{\prime }(t_{a})=\int_{\frac{-L_{y}}{2}}^{\frac{L_{y}}{2}}\rho_{0}(y)\{\exp\left[i(-\frac{BW}{2L_{y}}y^{2}+\frac{BW}{2}y+\gamma G_{a}yt_{a})\right]\\ \;*\exp\left[-i(-\frac{BW}{2L_{y}}y^{2})\right]\}dy \end{array}$$

where S′(t_a_) is the signal acquired at time t_a_ after removing the quadratic term that has the largest influence on the visualization of the focal point, *ρ*_0_(y) is the spin density at position *y* (Chen et al., 2013), L_y_ is the FOV size in the y direction, BW is the bandwidth corresponding to the Chirp pulse, BW = *γ*G_e_T_e_, G_a_ is the acquisition gradient, and T_e_ is the duration of the spatiotemporal encoding gradient pulse G_e_.

In order to obtain two-dimensional images, an inverse FT was applied only in the direction of the readout gradient in the k-space data to obtain a 2D image. The conjugate of the position-related quadratic term was used to compensate for the residual quadratic phase in the phase encoding direction of the 2D image, and coil channels combination was used to obtain the final 2D complex domain images.

## 3. Results

**Figure 1:**
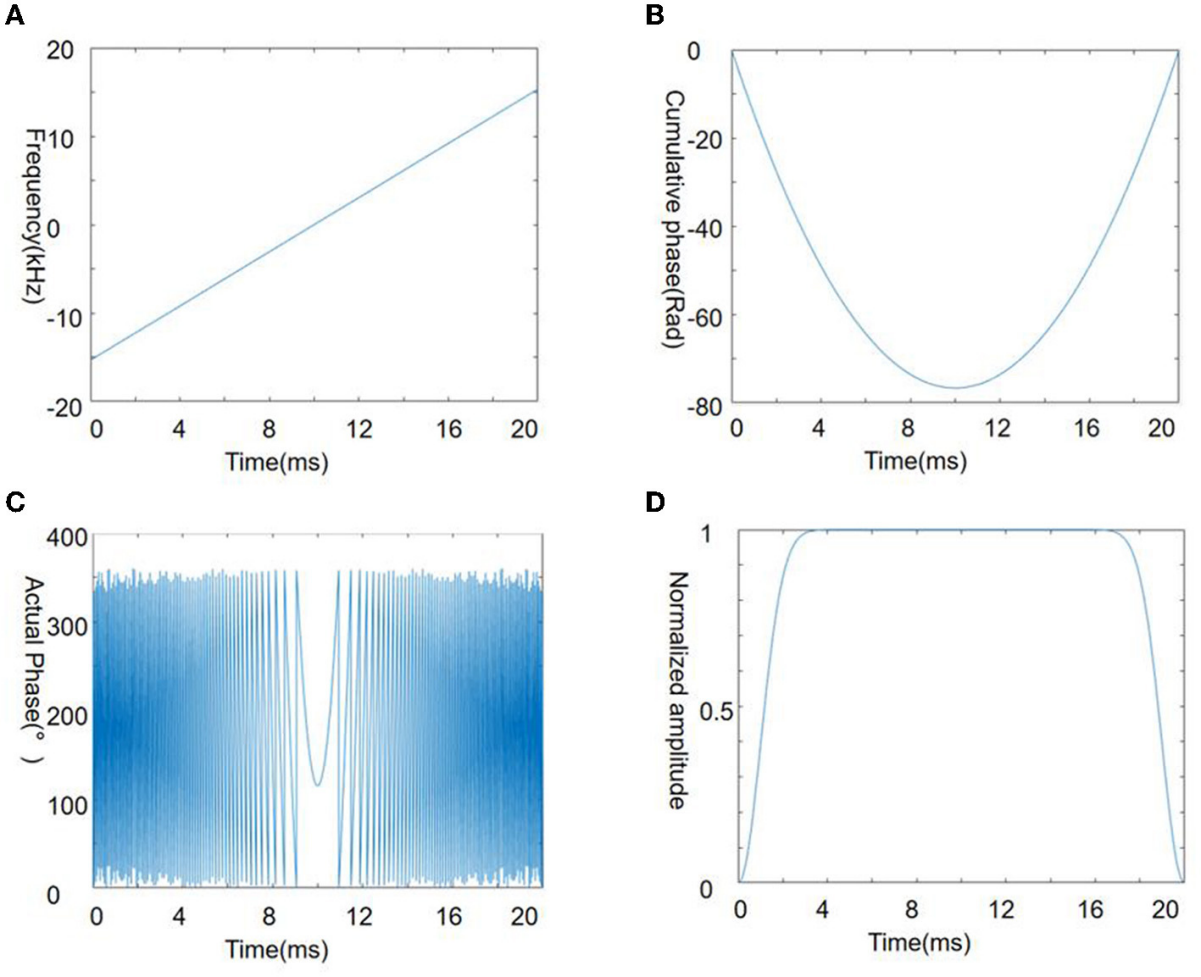
Chirp pulse used in the present study. Four panels illustrate the frequency **(A)**, cumulative phase **(B)**, actual phase **(C)**, and amplitude **(D)** of the chirp pulse.

Figure 1 shows the Chirp pulse parameters used in this study, and the excitation frequency of the chirp pulse increases linearly with time (Figure 1A), while the cumulative phase changes quadratically with time (Figure 1B). When the accumulated phase is wrapped around into 0–360°, the phase at the top of the parabola (i.e., the phase stable point) and nearby change gradually with time whereas it shows substantial oscillations in other regions (Figure 1C). This behavior is an important characteristic of the chirp pulse and a highly desirable aspect of the SPEN signal.

**Figure 2:**
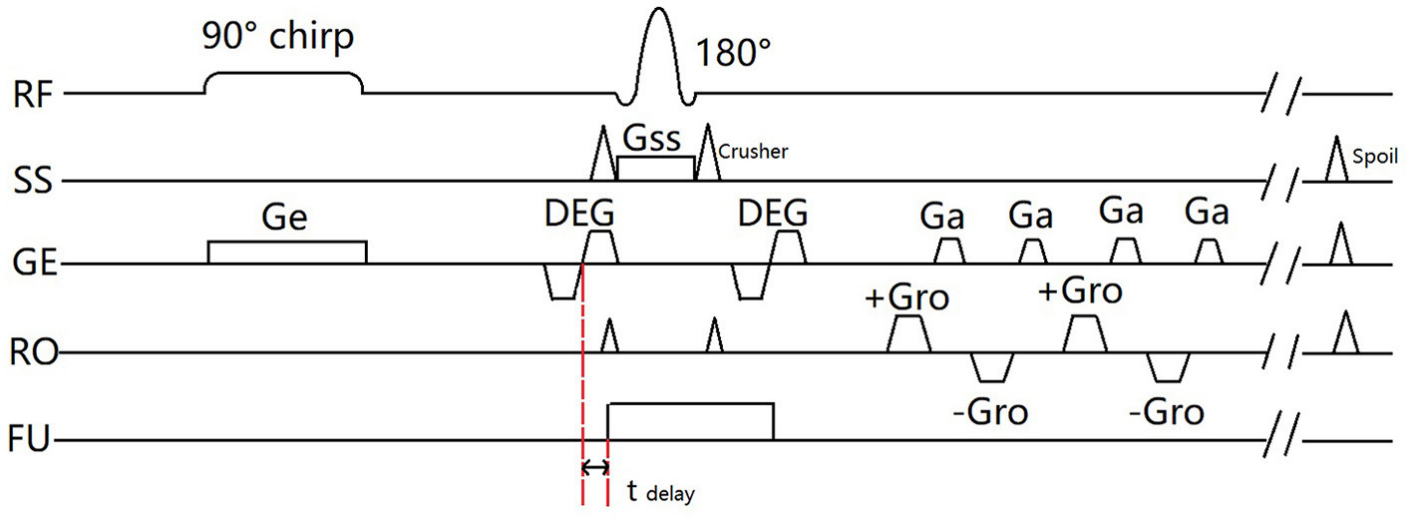
Timing diagram of the SPEN-ARFI sequence used in this study.

Figure 2 shows the SPEN-ARFI sequence used in the present study is the combination of a 90° chirp pulse and spatioatemporal-encoding gradient G_e_. A bipolar DEG is added on both sides of the 180° RF pulse. A delay exists in the synchronization between the triggering at the start of the second lobe of the DEG and application of the FU ARF pulse. The use of an EPI readout to collect k-space data means that an image can be acquired in one TR.

**Figure 3:**
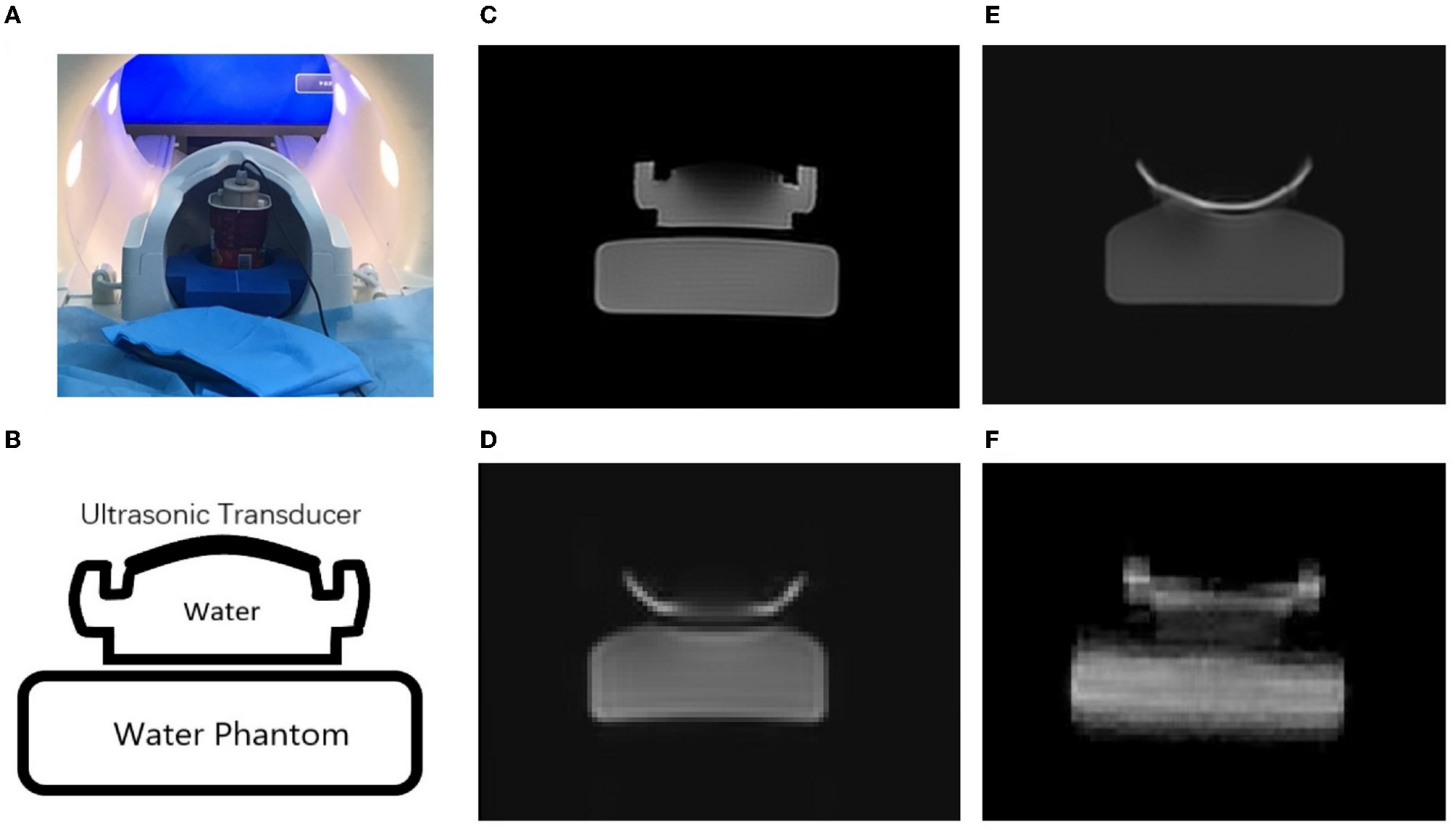
Experimental scenario of this study **(A)**. MR images obtained by using **(C)** FSE, **(E)** GRE-EPI, **(D)** SE-EPI and **(F)** SPEN sequences of the of ultrasound transducer and water phantom **(B)**.

Figure 3 shows a comparison of MR images acquired using different sequences, namely, FSE, GRE-EPI, SE-EPI, and SE-SPEN, of a water phantom with an ultrasonic transducer. The FSE image is considered as the reference against which the other images are compared. Figures 3A, B shows the ultrasound transducer was placed above the agar phantom inside the MRI system. As shown in Figures 3D, E, there are significant distortions in the SE-EPI and GRE-EPI images, especially near the transducer. In contrast, the SE-SPEN image as shown in Figure 3F exhibits only minor distortion.

**Figure 4:**
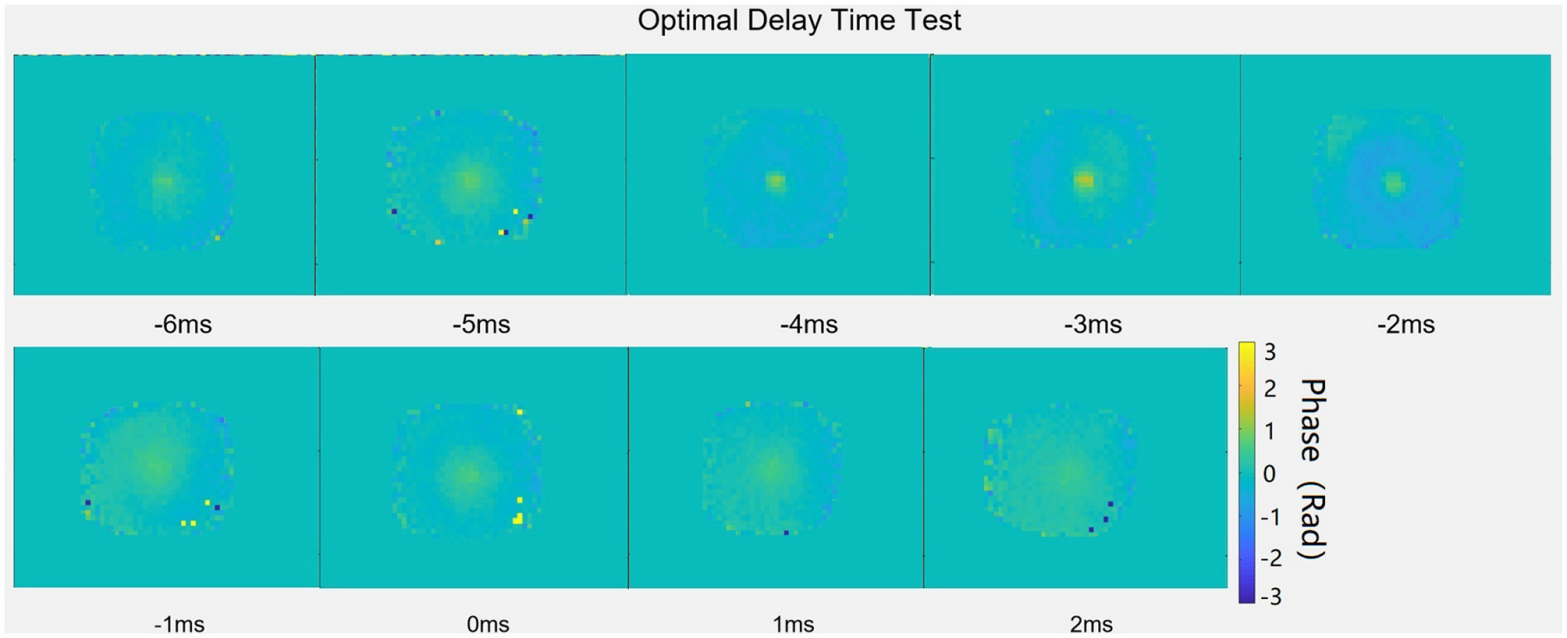
Effect of delay time (t_delay_) on phase contrast maps in an agar phantom.

As shown in Figure 4, several different delay times were investigated in the agar phantom, ranging from −6 to 2 ms, and the phase change and displacement were found to be greatest when the delay time was set to −3 ms, indicating that the ultrasound system should be turned on 3 ms before the DEG was applied.

**Figure 5:**
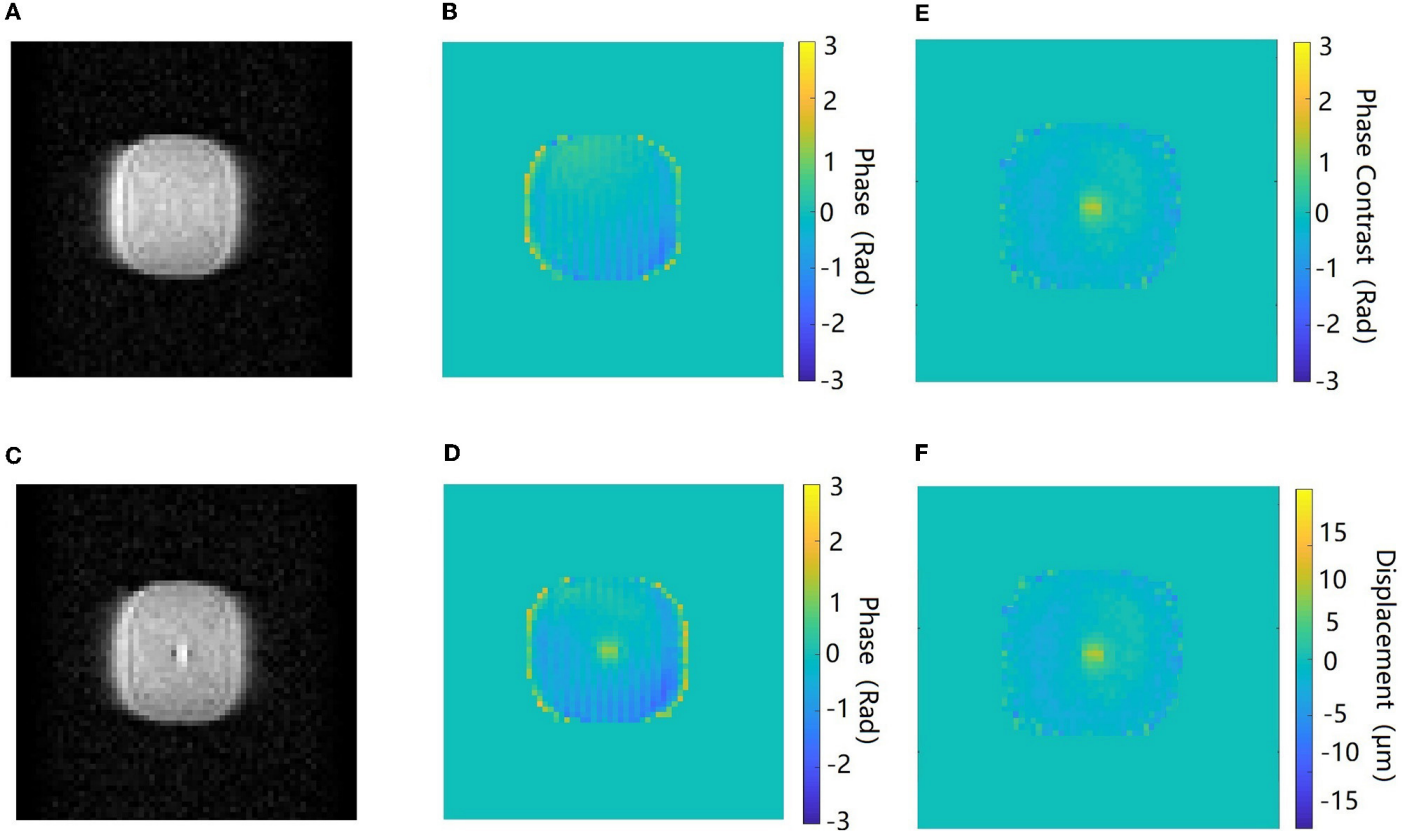
Amplitude and phase images acquired in the axial orientation before **(A, B)** and after **(C, D)** the ultrasound beam is applied to the target in the agar phantom. The phase contrast map **(E)** and the corresponding displacement map **(F)**. The color bar of the phase images and phase contrast map corresponds to a phase interval of π to –π.

**Figure 6:**
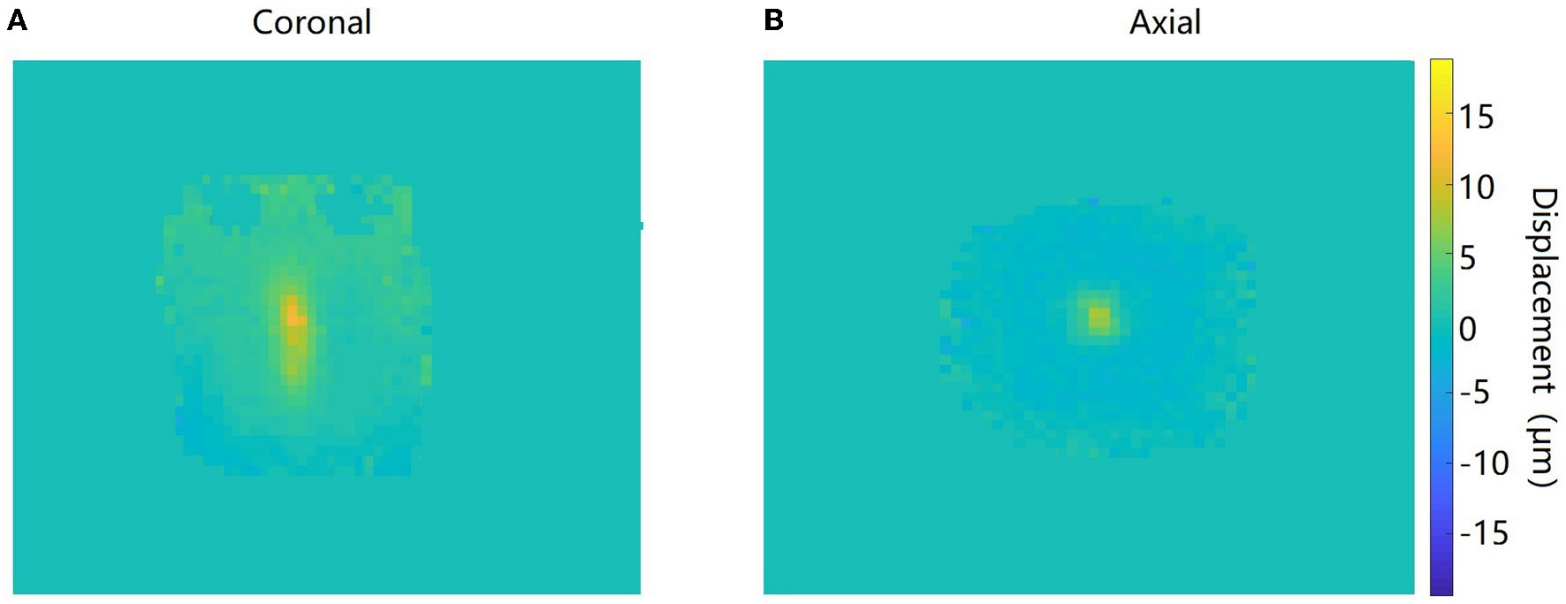
Displacement maps of the ultrasound target acquired in coronal **(A)** and axial **(B)** orientations in the *ex vivo* porcine brain.

Figure 5 shows the amplitude, phase, and the corresponding maps acquired in the axial orientation before and after the FU was applied to the agar phantom. The imaging parameters of the *ex vivo* porcine brain were kept consistent with the study of the agar phantom, and the displacement maps of an *ex vivo* porcine brain acquired in coronal and axial orientations are shown in Figure 6.

**Figure 7:**
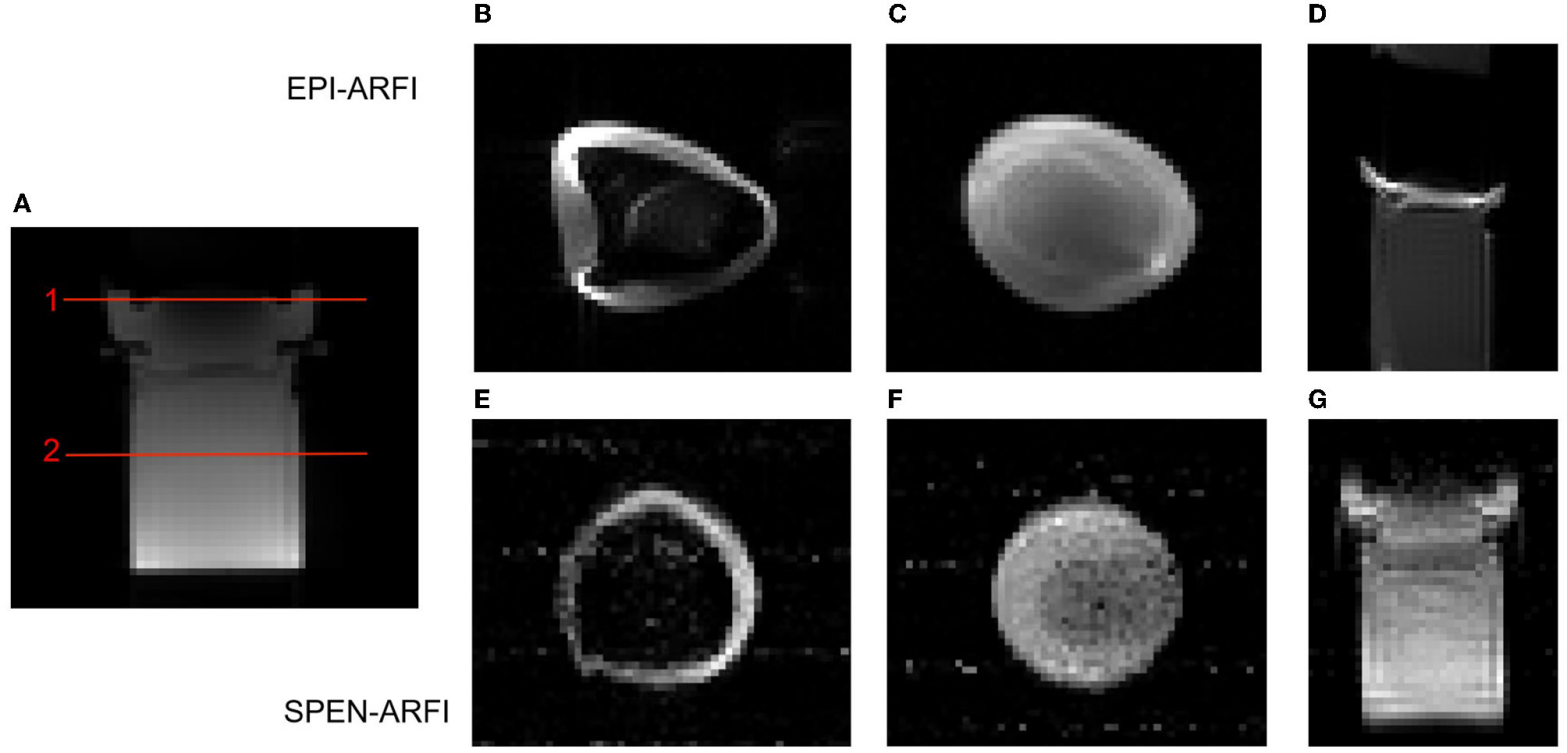
Comparison of EPI-ARFI and SPEN-ARFI sequences under inhomogeneous field. Red lines represent two different slices: Slice 1: **(B, E)**; Slice 2: **(C, F)**. **(A)** SE-ARFI in coronal. **(B, C)** EPI-ARFI in axial. **(D)** EPI-ARFI in coronal. **(E, F)** SPEN-ARFI in axial. **(G)** SPEN-ARFI in coronal.

The comparison results of SPEN-ARFI and EPI-ARFI under an inhomogeneous field are shown in Figure 7. The EPI-ARFI image had significant distortion and even aliased artifacts, while the SPEN-ARFI image showed better image quality obviously.

**Figure 8:**
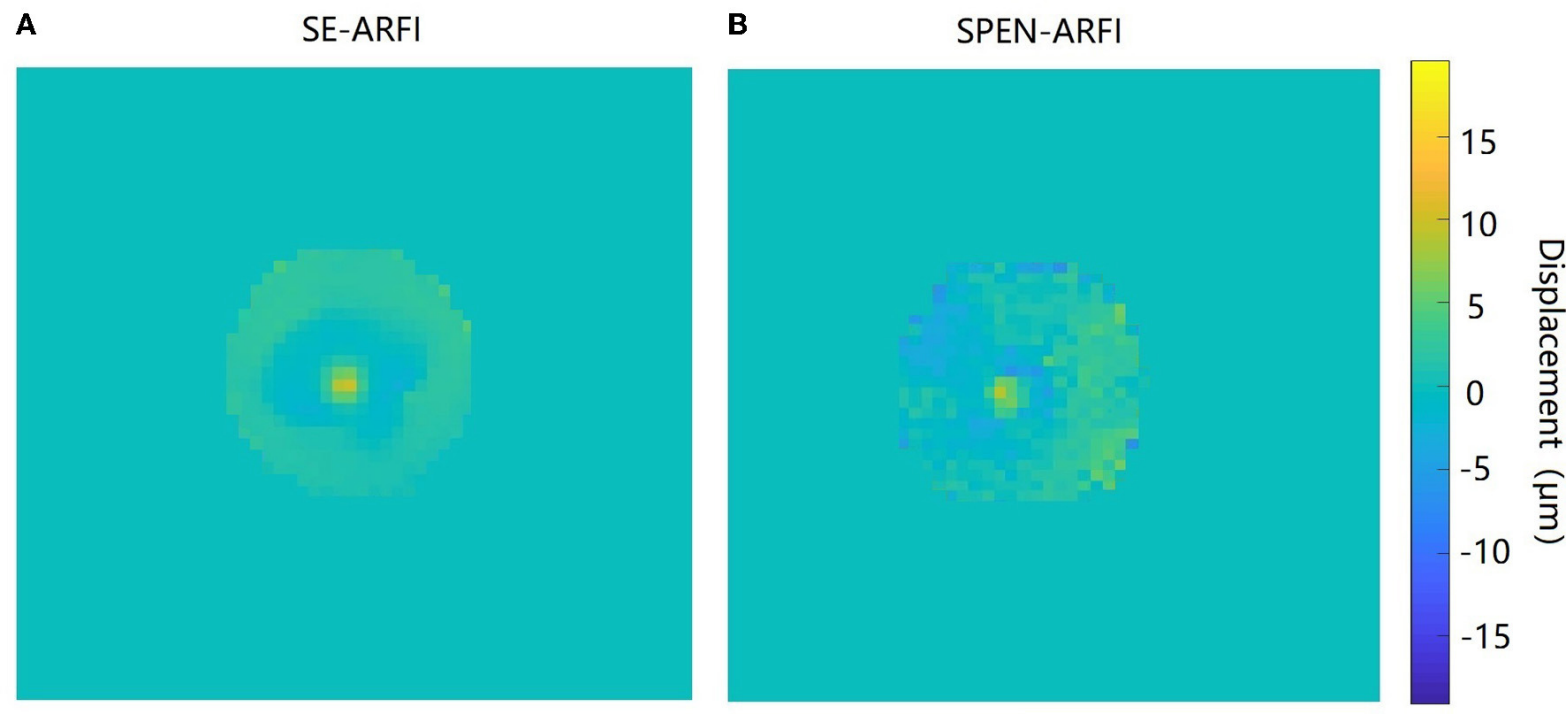
Comparison of SE-ARFI and SPEN-ARFI sequences. **(A)** Displacement map obtained by SE-ARFI. **(B)** Displacement map obtained by SPEN-ARFI.

The quantitative comparison experiment of the SPEN-ARFI and SE-ARFI sequence is shown in Figure 8. Finally, the average displacement at the focal point of the SPEN-ARFI sequence was 10.17 ± 0.69 μm and that of the SE-ARFI sequence was 9.52 ± 0.12 μm, indicating that the results of the comparative experiments were highly consistent, but the acquisition time of SPEN-ARFI was 250 ms, much faster than that of SE-ARFI (136 s).

## 4. Discussion

The addition of DEG and chirp pulse to a SE-EPI sequence has enabled the development of the SPEN-ARFI sequence, which has been used to monitor tissue displacement to an ARF pulse in both agar phantom and *ex vivo* porcine brain. The SPEN-ARFI technique offers several advantages over SE-ARFI and EPI-ARFI techniques. Compared with SE-ARFI, SPEN-ARFI greatly reduces scan time and thus minimizes ultrasound deposition during ARFI. Compared with EPI-ARFI, SPEN-ARFI has good resistance to magnetic field inhomogeneity, resulting in reduced image distortion and improved localization accuracy. This facilitates more precise positioning of the focus for FU stimulation and treatment, which is particularly relevant for ultrasound neuromodulation studies where metallic coatings, lead wires, paranasal sinus cavities, or metal implants produce large susceptibility artifacts. These factors can cause distortions and affect the quality of images obtained by traditional MRI pulse sequences, especially EPI sequences.

In the application of SPEN-ARFI, careful selection of Chirp pulses with the appropriate bandwidth is crucial. From Equation (4), it can be seen that a large bandwidth was chosen in this study, which allowed the SPEN-ARFI sequence to exhibit robustness against magnetic field inhomogeneities and obtain images with high spatial resolution albeit at the cost of reduced SNR in the images. Conversely, selecting a smaller bandwidth would diminish the benefits of SPEN-ARFI but improves the imaging SNR. Nonetheless, SPEN-ARFI has several limitations. Eddy currents can result in stripe artifacts on the phase map, as shown in Figures 5B, D, and the long TE results in the relatively poor image resolution and the decrease in SNR (Qiao et al., 2016). Additionally, the proposed SPEN-ARFI sequence has not been applied to *in vivo* animals due to the high cost of animal experiments and the need for sequence optimization such as reducing chirp pulse bandwidth and SAR value. To address these limitations, advanced SPEN image post-processing methods such as super-resolution image reconstruction can be applied to improve image spatial resolution and SNR (Ben Eliezer et al., 2010). In future works, SPEN-ARFI sequence needs further optimization for human application, to explore new applications of SPEN-ARFI in the field of ultrasound neuromodulation.

## 5. Conclusions

The present study presents a novel technique for acoustic radiation force imaging called the SPEN-ARFI sequence. Compared to conventional acoustic radiation force imaging methods, SPEN-ARFI offers superior imaging speed and enhanced tolerance toward magnetic field inhomogeneity. Overall, the SPEN-ARFI sequence represents a promising development in the field of acoustic radiation force imaging.

## Data availability statement

The original contributions presented in the study are included in the article/supplementary material, further inquiries can be directed to the corresponding author.

## Ethics statement

Ethical review and approval was not required for the animal study because this study does not involve investigations in human subjects or living animals (we just use an *in-vitro* porcine brain, commercially available at supermarkets) and so this did not need to be approved by the IRB and IACUC.

## Author contributions

JZ, HX, QH, and QG contributed to conception and design of the study. DK and XQ organized the database. XQ and YD did sequence tests. XQ wrote the first draft of the manuscript. JS, XH, and NR wrote some manuscripts. All authors participated in the revision, reading, and approval of the submitted version of the manuscript.
